# Moving towards environmental sustainability: information and communication technology (ICT), freight transport, and CO_2_ emissions

**DOI:** 10.1016/j.heliyon.2021.e08190

**Published:** 2021-10-19

**Authors:** Walid Chatti

**Affiliations:** Faculty of Economics and Administration, King Abdulaziz University, Jeddah, KSA, Saudi Arabia

**Keywords:** ICTs, Freight transport, Sustainable transportation, Environmental sustainability, GMM

## Abstract

This study explores the links between ICTs, transport, and CO_2_ emissions. Despite the harmful consequences of transport activity on the environmental quality, there is less scientific attention accorded to this major issue. In this regard, we explore the possibility of reducing environmental damages through the association of new technologies with freight transport activities (i.e. inland, rail, and air). The empirical technique based on 43 countries between 2002 and 2014 employs the 2-step system Generalized Method of Moments (GMM). Overall, the results are very ambitious confirming the ability of ICT in dampening pollution once it's well adapted in the transportation sector. First, the telephone and mobile phones are the most efficient technologies in terms of environmental sustainability when used in the rail and the inland transport sector, while the internet is best utilized in the air transport sector. Second, the telephone plays the role of an accelerator when interacting with intermodality to better improve the environment. Public policies and their implications are considered in the study.

## 1 Introduction

Transport systems actively contribute to the socio-economic development of countries. Freight transport essentially facilitates access to goods and materials, comprising the main distribution channels of imports and exports. However, freight activity is a major contributor to global atmospheric pollution, especially in the road transport sector, and if the average global temperature increases by 2 °C, the impacts are expected to be catastrophic for environmental quality (IPCC, 2014; McKinnon, 2016; Santos, 2017). According to the International Energy Agency (2016), transportation represents 30% of the EU's total GHG, of which road transport represented 72% in 2016. Despite relevant efforts made in other sectors of the economy, pollution has increased for the transport sector, and its eventual reduction appears prohibitively expensive because the system and the global economy as a whole is highly dependent on fossil fuel consumption and associated infrastructures (Uddin, 2012; Santos, 2017; Chatti et al., 2019). Therefore, both governments and transport companies have attempted to profit from some innovative solutions to dampen energy consumption, and thus reduce the environmental degradation associated with their activities.

Despite the key role that can be played by ICTs in reducing CO_2_ emissions, few studies have investigated the links between ICTs, freight activity, and the environment (Wang et al., 2015; Chatti, 2020; Centobelli et al. 2020a, 2020b). Few empirical papers explicitly show how ICTs reduce pollution when interacting with freight transport. The existing literature is focused in general on the identification of green practices and new technologies employed by industrial and service companies (Wang et al., 2015) but fails to identify the most efficient new technologies that can significantly decrease the environmental damages (Centobelli et al. 2020a, 2020b; Chatti, 2020). In addition, most studies have paid more attention to road freight transportation, neglecting the responsibility of other transportation modes in increasing pollution (e.g. air, rail, and inland).

This study aims to enrich the existent literature by exploring whether new technologies interact with freight transport to improve environmental quality with regards to carbon emissions reductions. The main contributions are presented as following. First, it aims to identify the most efficient technology that can reduce the negative effects of transportation activity (i.e. rail, inland, and air). Second, it sheds light on the necessity of combining both new technologies and multimodality (i.e. road-rail) as an ambitious solution for reducing pollution. Third, it attempts to explicitly propose an empirical analysis that quantifies the real effects of using new technologies in freight transport on the environment. Finally, it provides some practical policies in order to positively affect environmental quality for a global panel set comprising some developing and developed countries.

## 2 Literature review

The existing studies investigate the links relating ICTs to the environment and are classified into three groups. The first group demonstrates the deteriorating ICTs effects (Avom et al., 2020; Alatas, 2021), the second group shows the positive effects of ICTs (Ahmed and Le, 2021; Sahoo et al., 2021), and the third group suggests the conditional effects of ICTs (Majeed, 2018; Centobelli et al. 2020a, 2020b; Chatti, 2020).

The first group shows the environmental negative effects of ICTs. For example, Salahuddin et al. (2016) demonstrated the harmful impacts of using ICTs on sustainability in the long term. To do so, these authors considered a panel dataset for OECD countries between 1991 and 2012. They indicated that a 10% increase in internet adoption is able to rise carbon emissions by 1.6%. Another contribution is attributed to Avom et al. (2020) who confirmed the environmental hurting effects of ICTs considering some African economies. Similarly, Alatas (2021) investigates the negative impacts of ICTs on sustainability with regard to carbon emission augmentation.

Unlike these previous studies, the second group shows the environmental positive effects of ICTs. For example, Wang et al. (2015) exhibited how ICTs mitigate CO_2_ emissions generated by road freight transport. Based on case studies covering three UK grocery retailers, they found that ICTs improve environmental outcomes proposing some ways to decrease emissions through the reduction of energy consumption. Firstly, transport companies can optimize logistics operations by adopting advanced ICTs to reduce environmental damages caused by road freight transport environmental outcomes, given that 6% of atmospheric pollution is mainly caused by road freight transport (McKinnon, 2010).

The adoption of new technologies especially in logistic activities positively affects the profitability of companies. Centobelli et al. (2020a, 2020b) also highlighted the key role that can be played by green practices and ICTs in order to help companies acting in freight and logistics services. The use of innovative practices and new technologies in addition to other policies are able to reach sustainable development objectives for businesses (Húdik et al., 2019; Mangina et al., 2020; Skrúcaný et al., 2021).

Ozcan and Apergis (2018) underlined the environmental positive effects of ICTs using a sample of 20 developing economies between 1995 and 2015. In the same context, Lu (2018) confirmed the importance of using ICTs to mitigate environmental damages for Asian economies between 1993 and 2013. Similarly, Sahoo et al. (2021) reported the favorable impact of mobile phones and the internet in dampening carbon emissions in India between 1990 and 2018. The same positive effect was shown by Ahmed and Le (2021) during the period of time 1996–2017 including a set of Asian economies.

The third group shows conditional effects of ICTs. In this regard, Añón Higón et al. (2017) used a sample covering 142 countries between 1995 and 2010. They found that ICT can negatively affect environmental quality as a result of the increasing production of devices, ICT-related machines, and recycling of electronic waste. However, over the medium to long term, ICT can reduce carbon emissions by promoting smart cities, transportation networks, logistics network optimization, and energy consumption saving. This the case of some developed economies that have succeeded in reaching the required levels of ICTs penetration whereby undesirable effects are reduced significantly.

In the African case, Asongu (2018) examined the links between ICTs, globalization, and carbon emissions using 44 African countries over the period 2000–2012, exploring whether ICTs interact with globalization to improve environmental protection. Using the GMM methodology, the results showed the capability of new technologies in reducing the undesirable impacts of globalization. Similarly, Asongu et al. (2018) investigated the links relating ICTs to CO_2_. They measured ICTs in terms of internet and mobile phones adoption, and pollution in terms of CO_2_ emissions. The results showed that ICTs cannot reduce pollution once considering non-interactive estimations.

In the same context, Danish et al. (2018) attempted to clarify how ICTs can influence the environment through the interaction of ICTs with GDP and financial development. Using a set of panel models applied to emerging countries over the period 1990–2015, they found some interesting results: (i) ICTs, GDP and financial development positively affect CO_2_ emissions; (ii) the interaction of ICT with GDP is able to decrease environmental damages; and (iii) the association of ICT and finance negatively affects the environment. In the same context, Park et al. (2018) examined whether ICT, globalization and GDP affect environmental degradation using a sample of some European countries between 2001 and 2014. They found that ICT has a long-run relationship with environmental degradation. However, under some conditions, GDP and financial development can positively affect the environment.

In the same line, Chatti (2020) investigated whether ICT interacts with road freight transport to reduce pollution. He showed the negative impacts of ICTs on sustainability. However, the adoption of telephones and mobile phones in transport activity can reduce CO_2_ emissions. These new technologies are able to decrease carbon emissions by 2.26% and 0.85%, respectively. Indeed, ICTs can be considered a solution for reducing pollution, especially where interacting with road freight transport to increase energy efficiency. Other innovative practices, such as e-ticketing, smart transport, and reservations can help companies in order to better identify the most efficient combinations of routes and networks for more sustainable freight transport system (Waygood et al., 2013; Russo and Comi, 2012; Sarkan et al., 2017; Rybicka et al., 2018; Tsakalidis et al., 2020; Jereb et al., 2021).

Based on the above-discussed literature, it can be inferred that the conclusions provided by literature are conflictual since they report different effects of ICTs on environmental protection (positive, negative, and conditional effects). Indeed, the impacts of ICTs on the environment are conditioned by ICTs penetration levels, empirical methodology, sample, and time period used in the analysis. To the best of our knowledge, very few articles in the literature examined the role of new technologies through different modes of freight transport on sustainability. To fill this gap, this study explores the different impacts of ICTs on the environment over the period of time 2002–2014.

## 3 Empirical method

### 3.1 Data

We study whether ICT interacts with freight transport to improve environmental quality through the reduction of carbon emissions. To reach this goal, we employ balanced panel data comprising 43 countries between 2002 and 2014. The chosen economies and time frame are dictated by the availability of the dataset. The dependent variable is defined in terms of carbon emissions derived from liquid energy. ICTs are indexed by the variables internet, mobile phones, and fixed telephone technologies, as used by numerous previous researchers, including Asongu et al. (2019), Chatti (2020), and Chatti and Khoj (2020). We integrate also four control variables^1^.

Table 1 defines all dependent and independent variables introduced in the present study. These variables came principally from two sources, such as the World Bank and OECD. It also shows the different measurements used for the construction of each corresponding variable. The dependent variable refers to CO_2_ emission due to the consumption of petroleum fuel as a principal source. In this regard, the variables related to freight transport (i.e. rail, inland, and air) are measured in million ton-km while the technology adopted is measured per hundred inhabitants.

**Table 1:** Variable definitions.

Variables	Definitions and measurements
CO2liq	Carbon emissions derived from liquid fuel consumption (Kt)
INT	Internet users (per 100 inhabitants)
MOB	Mobile phone subscriptions (per 100 inhabitants)
TEL	Telephone landline subscriptions (per 100 inhabitants)
GDPg	Per capita gross domestic product growth rate (annual %)
POPg	Population growth rate (annual %)
REG	Regulation quality (estimate)
TO	Imports + exports of goods and services (% of GDP)
IFT	Inland freight transport (road/rail) in million ton-km
RFT	Rail freight transport in million ton-km
AFT	Air freight transport in million ton-km

Table 2 describes statistics of all variables integrated into the study. It reports that Estonia has the lowest level of carbon emission (817.741) while the United States of America has the maximum value of 2446414. Regarding ICT adoption, India belongs the minimum value of internet adoption (1.537) while Iceland is the most developed in terms of internet penetration (98.16). Moreover, India has the lowest value of telephone penetration while Switzerland has the highest value (74.616).

**Table 2:** Descriptive statistics.

	Obs.	Mean	S.D.	Min.	Max.
CO2liq	559	164066.3	372406.3	817.741	2446414
INT	559	57.173	25.4099	1.537	98.16
MOB	559	97.680	32.846	1.192	172.178
TEL	559	38.883	15.498	2.083	74.616
GDPg	559	2.488	4.505	-14.559	32.997
POPg	559	0.417	0.803	-2.258	2.890
REG	559	1.017	0.667	-0.706	1.970
TO	559	91.524	51.458	20.685	392.804
IFT	546	569113.4	1700150	660	1.26e+07
RFT	533	206012.6	604413	79	2946579
AFT	494	2979.86	6526.961	0	40617.74

*Note*: S.D. Standard Deviation. Obs. Observations. Min. Minimum. Max. Maximum.

Table 3 reports the correlation between CO_2_ emissions derived from liquid fuel (*CO2liq*) and ICTs (i.e. *INT*, *MOB*, and *TEL*) along with other independent variables. Intuitively, the results confirm a positive correlation between the telephone and the population growth, and carbon emission. However, mobile phones, the per capita GDP growth, and trade openness have a negative and significant correlation in relation to carbon emission. Here, it should be noted that the existence of multicollinearity issues is less significant when using interactive estimations (Brambor et al., 2006).

**Table 3:** Correlation matrix.

	CO2liq	INT	MOB	TEL	GDPg	POPg	REG	TO
CO2liq	1							
INT	-0.0203 (0.6317)	1						
MOB	-0.1503∗∗∗ (0.0004)	0.6385∗∗∗ (0.0000)	1					
TEL	0.1605∗∗∗ (0.0001)	0.5409∗∗∗ (0.0000)	0.1942∗∗∗ (0.0000)	1				
GDPg	-0.1192∗∗∗ (0.0048)	-0.4442∗∗∗ (0.0000)	-0.4057∗∗∗ (0.0000)	-0.3415∗∗∗ (0.0000)	1			
POPg	0.3143∗∗∗ (0.0000)	0.1572∗∗∗ (0.0000)	-0.0967∗∗ (0.0222)	0.1953∗∗∗ (0.0000)	-0.1576∗∗∗ (0.0002)	1		
REG	-0.0151 (0.7218)	0.7312∗∗∗ (0.0000)	0.4356∗∗∗ (0.0000)	0.6650∗∗∗ (0.0000)	-0.3886∗∗∗ (0.0000)	0.1826∗∗∗ (0.0000)	1	
TO	-0.5279∗∗∗ (0.0000)	0.2292∗∗∗ (0.0000)	0.3035∗∗∗ (0.0000)	0.0175 (0.6801)	0.0159 (0.7068)	-0.0098 (0.8179)	0.2133∗∗∗ (0.0000)	1

*Note:* P-values in parentheses. ∗∗∗Significant at 1%. ∗∗ Significant at 5%. ∗ Significant at 10%.

Table 4 shows the stationarity checks using two unit root tests which are developed by Levin-Lin-Chu (LLC, 2002) and Im et al. (2003). Despite the fact that the first unit root test (LLC, 2002) is less efficient for smaller samples, it considers the heterogeneity of sections. For the second unit root test (Im et al., 2003), its main advantage is dedicated to its ability to perform in small samples by considering the heterogeneity between them, whereby it eliminates serial correlation.

**Table 4:** Unit root tests.

	LLC test		IPS test	
	Level	Diff (1)	Level	Diff (1)
Ln CO2liq	-8.6422∗∗∗		-5.1210∗∗∗	
	(0.0000)		(0.0000)	
INT	-8.8060∗∗∗		-3.0935∗∗∗	
	(0.0000)		(0.0010)	
MOB	-7.2098∗∗∗		0.7854	-2.4949∗∗∗
	(0.0000)		(0.7839)	(0.0063)
TEL	-4.0727∗∗∗		3.0100	-4.5465∗∗∗
	(0.0000)		(0.9987)	(0.0000)
GDPg	-10.0562∗∗∗		-7.0887∗∗∗	
	(0.0000)		(0.0000)	
POPg	-20.0123∗∗∗		-2.6722∗∗∗	
	(0.0000)		(0.0038)	
REG	-5.4250∗∗∗		-4.6755∗∗∗	
	(0.0000)		(0.0000)	
TO	-8.1422∗∗∗		-5.3435∗∗∗	
	(0.0000)		(0.0000)	

*Note:* Estimated p-values are in parentheses.

∗∗∗ Significant at 1%. ∗∗ Significant at 5%. ∗Significant at 10%.

The acceptance of H0 means the non-stationarity of series, whereas the alternative hypothesis confirms the stationarity of different series. To decide between the acceptance and the rejection of H0, the p-value can be compared with the threshold of 10%. Considering the empirical specification related to ICTs, road freight transport, and CO_2_ emissions, the reported results confirm the stationarity of most variables except mobile phone and telephone adoption, which become stationary at the first difference. For the two other empirical specifications related to rail and inland freight transport, we find the same results in terms of stationarity. Concerning the empirical specification related to ICTs, air freight transport, and CO_2_ emissions, most variables are stationary, except air freight transport, which becomes stationary only at the first difference.

### 3.2 Empirical strategy

In order to understand how ICTs influence environmental quality, we employ the two-step GMM methodology as proposed by Chatti (2020). The empirical choice is motivated by five reasons: (i) the number of groups (n = 43) exceeds the time periods (t = 13); (ii) the dependent variable (*lnCO2liq*) does not change, given that the coefficient of first lag variable is larger than 0.8; (iii) the empirical investigation considers an eventual endogeneity bias, using instruments and time-invariant omitted variables; (iv) “inherent biases in the difference estimator are corrected with the system estimator” (Asongu, 2018); and (v) given that the econometric strategy employs panel dataset, differences across groups are considered in estimations.

The extension developed by Roodman (2009) is used for controlling the number of instruments, and consider any eventual dependence between sections^2^ (Boateng et al., 2016). The used two-step GMM strategy considers both equations in level and first difference (respectively).

$$lnCO2liq_{i,t}=\alpha_{0}+\alpha_{1}lnCO2liq_{i,t-r}+\alpha_{2}lnFT_{i,t}+\alpha_{3}ICT_{i,t}+\alpha_{4}ln{\left(ICT.FT\right)}_{i,t\;}+\overset{4}{\underset{n=1}{\sum }}\delta_{n}W_{n,i,t-r}+\gamma_{i}+\mu_{t}+\varepsilon_{i,t}$$

$$lnCO2liq_{i,t}-lnCO2liq_{i,t-r}=\alpha_{1}\left(lnCO2liq_{i,t-r}-lnCO2liq_{i,t-2r}\right)+\alpha_{2}\left(lnFT_{i,t}-lnFT_{i,t-r}\right)+\alpha_{3}\left(ICT_{i,t}-ICT_{i,t-r}\right)+\alpha_{4}\left(lnICT\cdot FT_{i,t}-lnICT.FT_{i,t-r}\right)+\sum_{n=1}^{4}\delta_{n}\left(W_{n,i,t-r}-W_{n,i,t-2r}\right)+\left(\mu_{t}-\mu_{t-r}\right)+\varepsilon_{i,t-r}$$

where $lnCO2liq_{i,t}$ is CO2 emissions derived from liquid energy for country *i* at year *t*, $\alpha_{0}$ is the constant, *lnFT*_*it*_ is the quantity of merchandise loaded by each transport mode (i.e. inland, rail, and air) for country *i* at year *t*, *ICT*_*i,t*_ is the communication technology (i.e. telephone, internet, and mobile phones) adopted in country *i* at year *t*, $\;ln{\left(ICT.FT\right)}_{i,t}$ indicates the interaction between ICT and urban freight transport for country *i* at year *t*, *W* incorporates four independent variables, such as per capita GDP growth, population growth, regulation quality, and trade openness, r equals one indicating the coefficient of autoregression, $\mu_{t}$ is the time-specific constant, $\gamma_{i}$ is the country effect, and $\varepsilon_{i,t}$ is the error term.

The dependent variable depends on a set of independent variables (i.e. *ICT*, *FT*, *POPg*, *GDPg*, *TO*, and *REG*). The main variables that can affect environmental quality have been highlighted by several studies (Omri et al., 2015; Chatti, 2020). Therefore, freight transport activity is expected to be associated with negative environmental effects (Wang et al., 2015; Saidi and Hammami, 2017; Chatti, 2020). The most egregious of these for macroeconomic analysis are carbon emissions (although localized air pollution is also a major issue), per capita GDP, and population growth, whereas regulation is expected to reduce pollution (Asongu et al., 2018).

## 4 Results and discussion

### 4.1 Results

Two specifications are considered in the estimations: without and with control variables. Within each empirical specification, we consider three different specifications in relation to freight transport. Moreover, each sub-specification is characterized in terms of different ICT technology. As used in several works, we utilize two tests to be sure that the empirical strategy is adequate: the test of AR(2) and the Hansen *J*-test. These tests inform us that H0 confirms the absence of correlation across instruments and error term, and the excluded instruments are not taken into account in the regressions. In addition, we show the Arellano and Bond (1991) test, namely AR(2), “where the null hypothesis (H0) indicates that the differenced errors are auto-correlated since the regression errors are not dependent and equally distributed” (Chatti, 2020, p. 129). The AR(2) test is “not robust” and is “weakened by instruments”, while the Hansen *J*-test is robust, but is also weakened by instruments. This latter is adopted to restrict the increase of instruments.^3^

Table 5 reports the findings related to ICTs, inland freight transport, and carbon emissions. In this estimation, we use only three control variables: population growth, regulation, and trade openness. The results show that ICTs positively affect carbon emissions, similar to findings reported by Asongu et al. (2019), Chatti (2020), Avom et al. (2020), Alataş (2021), and Su et al. (2021). This is due essentially to their great dependency on electricity consumption in relation to the provision of equipment and devices, and the use of related infrastructures. The contribution of ICTs to global CO_2_ emissions has been estimated to be 2% (Mingay, 2007).

**Table 5:** ICTs, inland transportation, and carbon emissions.

Variables	CO2liq					
	Inland Freight Transportation (IFT)					
	Without Conditioning Information			With Conditioning Information		
	MOB	INT	TEL	MOB	INT	TEL
Constant	0.913∗∗∗ (0.000)	0.427∗∗ (0.020)	0.624∗∗ (0.030)	0.919∗∗∗ (0.000)	0.602 (0.217)	0.612∗∗ (0.022)
Ln CO2liq (-1)	0.997∗∗∗ (0.000)	0.994∗∗∗ (0.000)	0.962∗∗∗ (0.000)	1.000∗∗∗ (0.000)	0.979∗∗∗ (0.000)	0.971∗∗∗ (0.000)
Internet		0.001∗ (0.096)			0.003 (0.141)	
Mobile	0.002∗∗∗ (0.004)			0.002∗∗ (0.021)		
Telephone			0.010∗∗ (0.024)			0.008∗ (0.076)
Ln IFT	0.230∗∗∗ (0.000)	0.127∗ (0.087)	0.341∗∗ (0.023)	0.227∗∗∗ (0.000)	0.201 (0.251)	0.294∗ (0.057)
Ln INT∗IFT		-0.127∗∗ (0.023)			-0.187 (0.169)	
Ln MOB∗IFT	-0.236∗∗∗ (0.001)			-0.239∗∗∗ (0.002)		
Ln TEL∗IFT			-0.302∗∗ (0.039)			-0.267∗ (0.063)
POP growth				0.020 (0.341)	0.034 (0.140)	0.026 (0.311)
Regulation				-0.002 (0.916)	-0.017 (0.518)	-0.015 (0.460)
Trade				0.0001 (0.700)	-0.00005 (0.839)	-0.0001 (0.606)
AR(2) test	(0.471)	(0.488)	(0.321)	(0.484)	(0.472)	(0.336)
Hansen *J*-test	(0.327)	(0.308)	(0.537)	(0.530)	(0.270)	(0.482)
Instruments	40	40	40	39	39	39
Groups	42	42	42	42	42	42
Obs.	504	504	504	504	504	504

P-values in brackets. ∗, ∗∗, ∗∗∗ significant at 10%, 5% and 1%, respectively.

However, the results show that the interaction between ICTs and inland freight transport (i.e. road-rail) negatively affects carbon emissions. The coefficient of -0.236 shows that environmental degradation can be reduced by 2.36% if the interaction *MOB∗IFT* improves by 10%. The coefficient of -0.127 implies that a 10% improvement in the interaction *INT∗IFT* is able to decrease pollution by 1.27%. It is worth noting that the interaction *TEL∗IFT* provides the most efficient and significant effect on environmental quality. Specifically, a 10% increase in the interaction *TEL∗IFT* decreases carbon emissions by 3.02%. In the same context, Llano et al. (2018) illustrated how the use of ICTs for multimodality^4^ can decrease carbon emissions. Compared with road transport, the use of ICTs in inland freight transport appears less harmful to the environment.

With the inclusion of control variables, mobile phones and telephone technologies seem to positively affect carbon emissions. The coefficients of 0.227 and 0.294 show that a 10% increase in *IFT* will increase environmental degradation by 2.27% and 2.94%, respectively. However, the interaction *MOB∗IFT* positively affects environmental sustainability. The value of -0.239 means that a 1% rise in the association *MOB∗IFT* decreases CO_2_ emissions by almost 0.4%. In addition, the interaction *TEL∗IFT* shows the same positive and significant effect on environmental quality with regard to pollution reductions. More specifically, a 10% increase in *TEL∗IFT* implies a 2.67% decrease in the pollution level. The results reinforce the association between multimodality and ICTs to facilitate data exchange and real-time visibility (Harris et al., 2015).

Table 6 reports the findings in relation to ICTs, air freight transport, and CO_2_ emissions. Based on the first empirical specification without control variables, air freight transport positively affects (i.e. increases) environmental damage. The magnitudes of 0.157, 0.107, and 0.198 imply that a 10% increase in air freight transport may increase carbon emissions by 1.57%, 1.07%, and 1.98%, respectively. However, the interactions *INT∗AFT*, *MOB∗AFT,* and *TEL∗AFT* seem to have negative effects on carbon emissions, which indicates that increasing ICT adoption in air freight transportation will accelerate its positive impact on environmental quality.

**Table 6:** ICTs, air freight transportation, and carbon emissions.

Variables	CO2liq					
	Air Freight Transportation (AFT)					
	Without Conditioning Information			With Conditioning Information		
	MOB	INT	TEL	MOB	INT	TEL
Constant	0.588∗∗ (0.034)	0.335∗∗ (0.035)	0.546∗ (0.075)	0.608∗∗ (0.031)	0.383∗ (0.075)	0.636 (0.231)
Ln CO2liq (-1)	0.990∗∗∗ (0.000)	0.993∗∗∗ (0.000)	0.993∗∗∗ (0.000)	0.990∗∗∗ (0.000)	1.003∗∗∗ (0.000)	0.999∗∗∗ (0.000)
Internet		0.001 (0.182)			0.003∗ (0.068)	
Mobile	0.001 (0.102)			0.001 (0.179)		
Telephone			0.005 (0.142)			0.006 (0.369)
Ln AFT	0.157∗∗ (0.041)	0.107∗∗ (0.069)	0.198∗ (0.093)	0.163∗ (0.054)	0.160∗ (0.071)	0.246 (0.289)
Ln INT∗AFT		-0.104∗ (0.079)			-0.163∗ (0.082)	
Ln MOB∗AFT	-0.153∗∗ (0.042)			-0.158∗∗ (0.054)		
Ln TEL∗AFT			-0.197∗ (0.097)			-0.250 (0.288)
POP growth				0.016 (0.279)	0.029∗ (0.051)	0.023 (0.259)
Regulation				-0.016 (0.424)	-0.018 (0.430)	0.006 (0.852)
Trade				0.00001 (0.935)	0.00003 (0.847)	0.00009 (0.734)
AR(2) test	(0.463)	(0.429)	(0.734)	(0.432)	(0.266)	(0.682)
Hansen *J*-test	(0.416)	(0.344)	(0.242)	(0.357)	(0.351)	(0.188)
Instruments	34	34	34	35	35	35
Groups	38	38	38	38	38	38
Obs.	456	456	456	456	456	456

P-values in brackets. ∗, ∗∗, ∗∗∗ significant at 10%, 5% and 1%, respectively.

Considering the second specification, it appears that air freight transport positively increases carbon emissions. A 10% increase in air freight activity may increase carbon dioxide emissions by 1.63% and 1.60%, respectively. This suggests that increasing air freight transport undermines environmental sustainability. However, the adoption of ICTs in air freight activity can reduce pollution. Indeed, the magnitudes of -0.158 and -0.163 note that a 10% improvement in *MOB∗AFT* and *INT∗AFT* leads respectively to 1.58% and 1.63% decreased pollution. These findings are largely supported by several authors who underlined the importance of using internet and mobile phone technologies to reduce CO_2_ emissions. Using efficient infrastructure networks, ICTs can reduce the need for transportation (see Gutierrez et al., 2009). The simple association of mobile phones with internet technology reduces physical meetings, and thus decreases urban pollution. In addition, the adoption of internet applications can be useful for companies’ competitiveness, particularly in the air transport sector (Buhalis, 2004; Wang et al., 2011; Agheli and Hashemi, 2018).

Table 7 presents the findings related to ICTs, rail freight transport, and CO_2_ emissions. The results broadly show the positive impact of *RFT* on carbon emissions, confirming its negative impact on the environment, reaffirming earlier studies (e.g. Asongu, 2019; Chatti, 2020). Specifically, the magnitudes of 0.157, 0.117, and 0.207 show that a 10% increase in rail freight transport may increase pollution by 1.57%, 1.17%, and 2.07%, respectively. However, the interaction between ICTs and rail freight transport seems to have a positive impact on environmental sustainability. Firstly, the coefficient of -0.111 reports that a 10% increase in *INT∗RFT* will decrease environmental damages by 1.11%. Secondly, a 10% increase in the interaction *MOB∗RFT* will reduce carbon emissions by 1.55%. Thirdly, the coefficient of -0.194 means that a 10% augmentation in the interaction *TEL∗RFT* will improve environmental quality by 1.94%. Moreover, the findings further suggest that the association *TEL∗RFT* is more efficient in terms of reducing environmental degradation than using the other technologies.

**Table 7:** ICTs, rail freight transportation, and carbon emissions.

Variables	CO2liq					
	Rail Freight Transportation (RFT)					
	Without Conditioning Information			With Conditioning Information		
	MOB	INT	TEL	MOB	INT	TEL
Constant	0.591∗∗∗ (0.001)	0.324 (0.106)	0.454 (0.127)	0.708∗∗ (0.049)	0.328 (0.619)	0.627∗ (0.061)
Ln CO2liq (-1)	0.994∗∗∗ (0.000)	0.994∗∗∗ (0.000)	0.986∗∗∗ (0.000)	0.969∗∗∗ (0.000)	0.991∗∗∗ (0.000)	0.975∗∗∗ (0.000)
Internet		0.001∗ (0.064)			0.002 (0.248)	
Mobile	0.001∗∗ (0.025)			0.001 (0.115)		
Telephone			0.006∗∗∗ (0.000)			0.006∗∗∗ (0.001)
Ln RFT	0.157∗∗∗ (0.001)	0.117∗∗ (0.018)	0.207∗∗∗ (0.000)	0.181∗∗ (0.03)	0.188 (0.202)	0.198∗∗∗ (0.001)
Ln INT∗RFT		-0.111∗∗ (0.021)			-0.164 (0.238)	
Ln MOB∗RFT	-0.155∗∗∗ (0.002)			-0.162∗∗ (0.010)		
Ln TEL∗RFT			-0.194∗∗∗ (0.003)			-0.191∗∗∗ (0.002)
POP growth				0.062∗∗ (0.027)	0.035 (0.239)	0.034 (0.329)
Regulation				-0.008 (0.602)	-0.011 (0.704)	-0.012 (0.697)
Trade				-0.0001 (0.671)	0.0001 (0.724)	-0.0002 (0.541)
AR(2) test	(0.456)	(0.485)	(0.282)	(0.474)	(0.494)	(0.296)
Hansen *J*-test	(0.433)	(0.237)	(0.458)	(0.411)	(0.311)	(0.309)
Instruments	34	34	34	39	39	39
Groups	41	41	41	41	41	41
Obs.	492	492	492	492	492	492

P-values in brackets. ∗, ∗∗, ∗∗∗ significant at 10%, 5% and 1%, respectively.

Relative to the second specification, the findings show that rail freight transport positively affects pollution. Specifically, the coefficients of 0.181 and 0.198 imply that a 10% increase in *RFT* is able to reduce emissions by 1.81% and 1.98% (respectively). However, the adoption of new technology in *RFT* can improve the environment, with the consideration of CO_2_ emissions reductions. The magnitudes of -0.162 and -0.191 show that the environmental quality will be increased by 1.62% and 1.91% (respectively) if the interactions *MOB∗RFT* and *TEL∗RFT* improve by 10%. Moreover, it is worth noting that the combination *INT∗RFT* does not affect the environment. The findings also illustrate that the adoption of telephone technology in *RFT* is more efficient in reducing pollution than utilizing the other new technologies. The use of ICTs is clearly of importance in the management of organizations and is seen as a key factor of the integration of supply chain and companies’ competitiveness (Cepolina and Ghiara, 2013; Molero et al., 2019).

### 4.2 Discussion

Based on the previous results, the adoption of ICTs positively influences carbon emission; thus, decreasing environmental protection. Despite the positive effect of ICTs in terms of reducing CO_2_ emission in some developed countries, it should be noted that the net effect is negative considering the global panel of countries. This study includes the most populated countries that have higher levels of carbon emissions, such as China, Mexico, Russia, Mexico, and India in addition to other developed countries. In the same line, the environmental harmful effects of ICTs is due to the fact that ICT penetration needs to reach a certain threshold level before realizing the desired effect. In this regard, Asongu et al. (2018) suggested that the threshold level is 150% and 42.5% for mobile phones and internet penetration (respectively). Those authors considered a panel dataset of 44 economies in sub-Saharan Africa. Therefore, the required threshold levels are different between countries dependent to the development degree and the adoption of ICTs in each economy.

Another interesting finding is related to the importance of the complementarity between ICTs and freight transport activity in order to positively affect environmental quality. Despite its unexpected impact, the interaction between new technologies and the transport sector is of importance to dampen significantly the CO_2_ emissions due to the freight transport activity (i.e. inland, rail, and air). This unexpected effect came from the fact that ICTs and freight transport negatively influence environmental quality but there is no direct evidence about their impact once associated together. This effect may be different when considering heterogeneity across countries since this modeling framework does not consider the specificities of each country to avoid the endogeneity issue (Asongu et al., 2018). However, this important result is in perfect accordance with previous research works that were proposed by Mckinnon (2016), Li and Yu (2017), and Centobelli et al. (2020a). Those authors confirmed the possibility of reducing pollution when using ICTs in freight transportation.

Another lesson can be deduced from the determination of the most efficient ICT that should be adopted considering the specificity of each transportation sector (i.e. rail, air, and inland). In this regard, policymakers may have a clear idea about which new technology is appropriate to positively affect environmental sustainability. Despite the crucial importance of using telephone networks in dampening CO_2_ emission, especially for rail and inland freight transport, the telephone penetration rate is becoming very low for most countries over the last years. Chatti (2020) reported the positive impact of telephone and mobile phones technologies on environmental quality when interacting with road freight transportation. Moreover, the telephone appears to be the most efficient technology for environmental protection. For example, a 10% improvement in the association between telephone and road transportation is able to decrease pollution by 2.23%. Regarding air freight transport, the use of internet networks by firms and businesses can help in reducing carbon emissions.

Finally, this study has the advantage of shedding some light on the importance of applying ICT for intermodality (road-rail). In practice, it is well known that intermodality is less harmful to the environment but there is no explicit study that measures this real effect once associated with ICT. To avoid this empirical insufficiency, the study not only confirms the benefits of interacting ICT with intermodality on environmental protection but also shows its accelerator role towards environmental quality. This positive impact is fully strengthened using the telephone technology in intermodality. Llano et al. (2018) showed the importance of intermodality on sustainability in Spain. Promoting intermodality in freight transport appears to be a good solution for more efficient and sustainable transport systems.

## 5 Conclusion

This study examined the interactive association relating ICTs to freight transport to influence CO_2_ emissions over the period 2002–2014. ICT refers to the internet, mobile phones, and telephone penetration levels, while freight transport is approximated in terms of rail, inland, and air freight transport. Using the GMM approach, the results suggest some interesting findings: (i) the only use of ICTs and freight transport increase CO_2_ emissions; (ii) the interaction between ICTs and freight transport can improve environmental quality with regards to carbon emissions reductions; (iii) the interactions of telephone and mobile phone technologies with rail, and inland freight activities are more efficient in dampening environmental degradation than adopting internet technology; (iv) the interaction between telephone and multimodality (i.e. road-rail) can significantly accelerate environmental quality; and (v) the use of the internet is the most efficient technology in reducing CO_2_ emissions where interacting with air freight transport.

### 5.1 Policy implications

The findings also further suggest the important role that can be played by ICTs in dampening the environmental hurting effects of freight transport activity, which is an egregious cause of pollution. Empirically speaking, a 10% improvement in the association between new technologies and freight transportation can reduce environmental degradation by between 1.3% and 3%. Therefore, both policymakers and transport companies could fully profit from the implementation of new ICT solutions for logistics and urban freight transport. These efficient technologies would be useful to facilitate the management, planning, and supply chain applications during the spatial movement of goods. Moreover, the adoption of ICTs in urban freight transport is able to reduce numerous urban costs; thus, limiting atmospheric pollution, delays, and accidents within urban areas.

The findings also suggest the importance of adopting ICTs in multimodal transport to reduce pollution. More specifically, the only interaction between telephone technology and inland freight transport (road–rail) can reduce carbon emissions by between 2.39% and 3.02%. First, there is an opportunity for policymakers to be focused more on the integration of new technologies with intermodality to accelerate change toward environmental sustainability. Second, governments should develop telephone technology rather than the internet and mobile phones when used with multimodal transportation.

Finally, this empirical research is the first to explicitly identify the capability of ICTs in reducing environmental degradation when interacting with various modes of transport (rail, inland, and air). This paper also highlights the necessity of applying the appropriate new technology, dependent on each specific mode of transport. In this regard, policymakers are invited to choose the appropriate technology that realizes the desired impact in terms of reducing pollution. For example, internet technology is better used in the air transportation sector while telephone and mobile phones are more profitable with rail and inland freight transportation.

### 5.2 Limitations

Like most research works, this study has some limitations that should be considered in the future. First, it neglects the existence of heterogeneity between countries. The environmental profitable effects of ICT could be different when considering developing, emerging, and developed countries. Second, this study considers only the freight transport activity but it could be profitable to include both passenger and freight transport in the estimations. Third, this study takes into account only one measure of carbon emissions such as CO_2_ emission from liquid fuel.

### 5.3 Future extensions

In the future, we plan to consider the heterogeneity across developing and developed countries as proposed by Majeed (2018). Indeed, the interaction between ICTs and freight transport would have different effects on the environment, dependent on the development level for the corresponding group of economies. Moreover, it would be interesting to examine how some new technologies interact with passenger transport activities to reduce pollution, taking into account other pollution indicators (e.g. CO_2_ intensity, per capita CO_2_ emissions, etc.).

## Declarations

### Author contribution statement

All authors listed have significantly contributed to the development and the writing of this article.

### Funding statement

This research did not receive any specific grant from funding agencies in the public, commercial, or not-for-profit sectors.

### Data availability statement

Data will be made available on request.

### Declaration of interests statement

The authors declare no conflict of interest.

### Additional information

No additional information is available for this paper.

## Footnotes

1 We include three control variables in the estimations (Tables 5, 6, and 7), such as population growth, regulation, and trade openness.

2 Baltagi (2008).

3 To avoid the proliferation of instruments, the total instruments in chosen sub-empirical specifications should be less than the total of countries.

4 Multimodality is defined as transportation activity using at least two modes at the same time. Intermodality can be considered as a particular type of multimodality which utilizes the same loading unit, such as a shipping container.
